# Heterogeneous Lineages of DNA Transposons Encode a TET/JBP Dioxygenase in Fungi

**DOI:** 10.3390/biology14121741

**Published:** 2025-12-04

**Authors:** Kenji K. Kojima

**Affiliations:** Genetic Information Research Institute, East Palo Alto, CA 94303, USA; kojima@girinst.org

**Keywords:** TET/JBP dioxygenase, DNA methylation, *ESTA*, *Plavaka*, transposon silencing, repeat-induced point mutation (RIP)

## Abstract

Epigenetic DNA modification is a key component of the defense system against invading nucleic acids such as transposons. TET/JBP dioxygenases oxidize 5-methylcytosine and lead to its replacement by cytosine in mammals. Expansion of *TET/JBP* genes and their association with DNA transposons were previously reported in Basidiomycota fungi. In this study, a thorough bioinformatics investigation of *TET/JBP* genes revealed that diverse groups of DNA transposons have captured a TET/JBP dioxygenase in three lineages of fungi: Pucciniomycetes (rusts) and Agaricomycetes (mushrooms) in Basidiomycota, and Pezizomycetes (morels and truffles) in Ascomycota. *TET/JBP* genes encoded by DNA transposons can be classified into three types, designated as PU, AG, and PE here. The PU type is distributed in Pucciniomycetes and encoded by seven different lineages of DNA transposons (*Dileera*, *hAT*, *Harbinger*, *IS3EU*, *EnSpm*, *ESTA*, and *Helitron*). The AG type is distributed in Agaricomycetes, and encoded by two lineages of DNA transposons (*Kyakuja* and *Zisupton*). The PE type is distributed in Pezizomycetes and Agaricomycetes, and encoded by five lineages of DNA transposons (*Zisupton*, *IS3EU*, *EnSpm*, *Plavaka*, and *Helitron*). Phylogenetic analysis indicated several transmission events from certain DNA transposon lineage to another. These transposon-encoded TET/JBP dioxygenases likely contribute to the escape of transposons from the methylation-based silencing system in fungi.

## Simple Summary

The methylation of cytosine in DNA is an epigenetic mark regulating gene expression and silencing transposons. TET/JBP dioxygenases are the only known group of enzymes that contribute to remove methylcytosine from DNA. It is known that some fungi encode multiple *TET/JBP dioxygenase* genes, often near transposase genes of DNA transposons. In this study, it is confirmed that diverse groups of DNA transposons encode a TET/JBP dioxygenase. *TET/JBP dioxygenase* genes were encoded by 11 different lineages of DNA transposons in 3 lineages of fungi: Pucciniomycetes (rusts), Agaricomycetes (mushrooms), and Pezizomycetes (morels and truffles). *TET/JBP* genes are transferred between different DNA transposons. Transposons encoding a TET/JBP dioxygenase likely escape from transposon silencing by removing epigenetic marks.

## 1. Introduction

Epigenetic DNA modification is the key regulator of gene expression as well as the key component of the defense system against invading nucleic acids such as viruses and mobile genetic elements [1,2]. Evolutionary arms-race between prokaryotes and phages results in diverse mechanisms of modification of DNA bases and backbones [2,3,4,5]. Compared to prokaryotes, eukaryotes have limited types of DNA modification [6]. The dominant modified base in eukaryotes is 5-methylcytosine (5mC).

In eukaryotes, DNA methylation and histone modification regulate the gene expression of transposons [7]. As a counteraction, some transposons encode an enzyme directly modifying epigenetic marks or a regulator controlling epigenetic modifications. Plant *Vandal* families of *MuDR* DNA transposons encode a protein regulating their own epigenetic silencing in a sequence-specific manner [8]. *SET histone methyltransferase* genes are incorporated by several different transposon lineages, such as *HarbingerS* and *MuDR* families from the oomycete *Phytophthora* [9,10].

The TET/JBP family of dioxygenases are the only known group of enzymes that actively modify the methyl group of 5mC [11]. Metazoan Ten-eleven translocation (TET) proteins oxidize 5mC to 5-hydroxymethylcytosine (5hmC) and other oxidized forms, and these oxidized cytosine derivatives are finally replaced by unmodified cytosine through base excision repair machinery. J-binding proteins (JBP) encoded in some kinetoplastids catalyze hydroxylation of thymine, which is further glycosylated to become base J, beta-D-glucopyranosyloxymethyluracil [12].

Eukaryotic transposons are traditionally grouped into two groups: retrotransposons and DNA transposons [13]. Eukaryotic DNA transposons are classified into 24 “superfamilies” in Repbase (https://www.girinst.org/repbase/ (accessed on 21 March 2019)) [14,15]. Basically, there is little sequence similarity between superfamilies; however, even different eukaryotic transposon superfamilies often share conserved motifs inside of their “transposase” proteins. Most superfamilies encode a transposase protein which conserves three acidic residues (DDD or DDE) for the DNA strand transfer reaction [16]. *EnSpm* (also called *CACTA*), *Mirage*, *Chapaev*, and *Transib*, all share C(2)C and H(3-4)H motifs between the second D and the last E catalytic residues, and the term *CMC* was introduced to represent the lineage of *EnSpm*, *Mirage*, and *Chapaev* [17]. These motifs are also conserved among recombination-activating gene 1 (RAG1), which originated from *Transib* [18]. The *Helitron* superfamily encodes an HUH nuclease/Y1 transposase and a helicase for their single-stranded transposition [19,20].

The number of *TET/JBP* genes is extremely high in some genera of fungi, such as *Laccaria* or *Coprinopsis*. Fungal *TET/JBP* genes appear associated with several different families of transposase genes [21,22]. The three groups of transposase genes were designated as *Zisupton*, *Dileera*, and *Kyakuja* and, together, are called *KDZ*. Another group of transposase genes associated with TET/JBP genes was reported as *Plavaka*, but *Plavaka* shows no sequence similarity to the other three transposase groups. Among them, only *Zisupton* is well characterized as a DNA transposon with terminal inverted repeats (TIRs) and target site duplications (TSDs) [23]. *Zisupton* generates 8 bp TSDs [14,23]. The close association between transposase and *TET/JBP* gene suggest that they co-mobilize as a transposon. However, it is unknown whether these *TET/JBP* genes reside within complete transposon structures and how widely they are distributed among fungal transposons.

Here, the complete transposon sequences that include a *TET/JBP dioxygenase* gene were characterized. *TET/JBP dioxygenase* genes were encoded by 11 different lineages of DNA transposons (*Zisupton*, *Kyakuja*, *Dileera*, *hAT*, *IS3EU*, *Harbinger*, *EnSpm*, *ESTA*, *Plavaka*, *Helitron1*, and *Helitron2*) in 3 lineages of fungi (Pucciniomycotina, Agaricomycotina, and Pezizomycotina). Phylogenetic and sequence analysis indicated the frequent but passive transmission of *TET/JBP* genes between different lineages of transposons. The possible functions of TET/JBP dioxygenases encoded by transposons along with fungal DNA methylation systems are discussed.

## 2. Materials and Methods

### 2.1. Characterization of TET^+^ Transposons (Transposons Encoding a TET/JBP Dioxygenase)

Censor [24] searches were performed against Repbase (as of 26 December 2017) [14] (https://www.girinst.org/repbase/) with the sequences of *TET/JBP dioxygenase* gene-associated proteins reported in [21] as queries. All Censor searches were performed with default parameters. *Zisupton-1_PGr* showed the significant similarity to some of these proteins (XP_001840494, XP_001876852, and EFX64608). The structure-based alignment by HHpred indicated the presence of *TET/JBP dioxygenase* gene in *Zisupton-1_PGr* [25].

Censor [24] searches were performed against the fungal genomes with the sequences of TET/JBP dioxygenases reported in [21] and the protein sequences encoded by *Zisupton-1_PGr.* Censor hits were extracted and clustered with BLASTCLUST 2.2.25 in the NCBI BLAST package with the thresholds at 75% length coverage and 75% sequence identity. The consensus sequence for each cluster was generated with the 50% majority rule applied with the help of homemade scripts. Censor searches were performed with the consensus sequence of each cluster against the genome. Up to 10 Censor hits over 80% sequence identity to the consensus were extracted with 5000 bp flanking sequences at both sides. Consensus sequences were regenerated to be elongated until they reached both termini. The termini were determined primarily based on the clear alignment borders and their adjacent TSDs. If they were not present, the TIRs and the similarity to other reported transposons were taken into consideration to determine the termini. The classification of transposons was performed based on the sequence homology to the reported transposons in Repbase [14]. The proteins are predicted with the help of Softberry FGENESH [26]. The characterized protein sequences of transposons were used as queries for the detection of more transposons encoding a TET/JBP dioxygenase.

All transposon sequences characterized in this study are available in the Supplementary Materials (Table S1 and Data S1) and are also submitted to Repbase [14].

### 2.2. Protein Sequence Alignment and Phylogenetic Analysis of Transposases

All nucleotide sequences of *hAT*, *Harbinger*, and *ISL2EU* sequences were extracted from Repbase [14] as of 27 December 2018 (https://www.girinst.org/repbase/). All sequences of *EnSpm*, *Chapaev*, *Mirage*, and *Transib* were extracted from Repbase as of 21 March 2019. All sequences of *Zisupton* and *IS3EU* were extracted from Repbase as of 19 March 2019. Protein sequences of ISs belonging to the IS*5* family were extracted from ISfinder [27] as of 19 March 2019 (https://isfinder.biotoul.fr/). The protein sequences of RAG1 from humans (NP_000439.1), zebrafish (NP_571464.1), and chimera (XP_007886047.1) were obtained from the NCBI Protein dataset (https://www.ncbi.nlm.nih.gov/protein/ (accessed on 19 March 2019)). The sequences of transposons characterized in this study were added to the respective dataset. All sequences were translated in six frames. Only the longest protein in each frame was collected and the proteins shorter than 300 residues were excluded in the analysis. If Softberry FGENESH [26] could predict the exon–intron structure to generate a protein-coding sequence that is better aligned with other transposase proteins, the predicted protein sequence was used.

The protein sequences were first aligned by MAFFT v.7.407 with the default parameters [28]. The presence of transposases was manually confirmed based on the alignment, and the protein sequences that do not correspond to transposases were removed from the analysis. The proteins that lack a substantial portion of the aligned region were also excluded. The final protein dataset was re-aligned by MAFFT with the linsi option. Because the proteins of *Kyakuja*, *Dileera*, and *Zisupton* were too diverged, Gblock, with less stringent options implemented in SEAVIEW 5.0.5 [29], was used to extract the residues aligned properly for the phylogenetic analysis.

Maximum-likelihood trees were generated at the PhyML 3.0 server (http://www.atgc-montpellier.fr/phyml/ (accessed on 24 April 2021)) [30] with 100 bootstrapping supports for the phylogenetic analysis of the superfamilies *EnSpm*, *Mirage*, *Chapaev*, *Transib*, *ESTA*, *Plavaka*, *Lanisha*, and RAG1, of the superfamilies of *Harbinger* and *ISL2EU* and the IS*5* family of ISs (*PHIS*), and of the group of *Kyakuja*, *Dileera*, and *Zisupton* (*KDZ*). Maximum-likelihood trees were generated at the PhyML 3.0 server (http://www.atgc-montpellier.fr/phyml/ (accessed on 19 November 2025) [30] with approximate likelihood-ratio test for branches (aLRT) values for the phylogenetic analysis of the *hAT* superfamily. The substitution model LG + G + I + F was used based on the Akaike Information Criterion (AIC) in all phylogenetic analyses except for *KDZ*. The substitution model WAG + G + F was used in the analysis of *KDZ*. The phylogenetic tree was rooted at the midpoint and visualized with FigTree v.1.4.3 (http://tree.bio.ed.ac.uk/software/figtree/ (accessed on 26 November 2018)).

### 2.3. Protein Sequence Alignment and Phylogenetic Analysis of TET/JBP Dioxygenases

The protein-coding sequences showing similarity to TET/JBP dioxygenases were manually extracted from transposon consensus sequences (Table S1). If Softberry FGENESH [26] could predict the exon–intron structure to generate a protein-coding sequence that is better aligned with other TET/JBP dioxygenases, the predicted protein sequence was used. Even though a methionine is usually observed at the *N*-terminal of the TET/JBP domain, the sequences without an *N*-terminal methionine were also included in the analysis to avoid the loss of information caused by the errors in the consensus reconstruction or in the exon–intron structure prediction.

The protein sequences were aligned by MAFFT v.7.407 with the linsi option [28]. The proteins that lack a substantial portion of the aligned region were manually excluded.

Maximum-likelihood trees were generated at the PhyML 3.0 server (http://www.atgc-montpellier.fr/phyml/ (accessed on 15 May 2021) [30] with 100 bootstrapping supports. The substitution model LG + G + I + F was used based on the Akaike Information Criterion (AIC) in the phylogenetic analyses of PU and AG type, and LG + G + I was used in the phylogenetic analysis of PE type. The phylogenetic tree was rooted at the midpoint and visualized with FigTree v.1.4.3 (http://tree.bio.ed.ac.uk/software/figtree/ (accessed on 26 November 2018)).

## 3. Results

### 3.1. DNA Transposons Encoding a TET/JBP Dioxygenase in Pucciniomycotina, Basidiomycota

#### 3.1.1. Dileera in KDZ (Kyakuja-Dileera-Zisupton)

**Figure 1:**
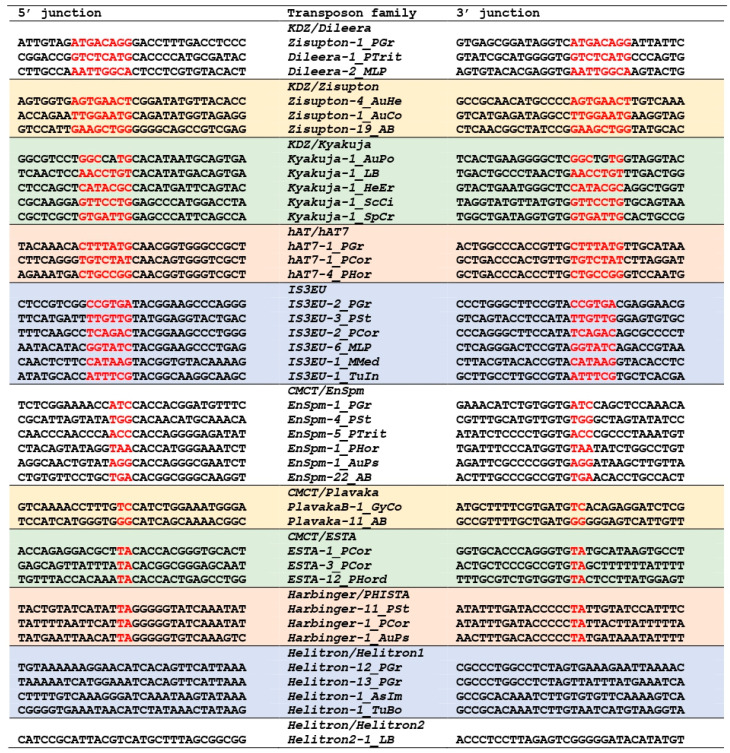
Termini and target site duplications (TSDs) of TET^+^ DNA transposons. One representative insertion is shown for each family. TSDs are colored in red. Background colors are just for visualization purposes. Species abbreviations in TE family names are shown in Table S1.

The comparison between the protein dataset reported in Iyer et al. [21] and the entries in Repbase [14] revealed that *Zisupton-1_PGr* from *Puccinia graminis* represents a lineage of *Dileera* encoding a TET/JBP dioxygenase. This is consistent with the report that the main association with *TET/JBP* genes in *Puccinia* and *Melampsola* is with *Dileera* among *KDZ* [21]. *Zisupton-1_PGr* generates 8 bp TSDs (Figure 1). Using the *TET/JBP dioxygenase* in *Zisupton-1_PGr* as queries for homology search, systematic screening of DNA transposons with *TET/JBP dioxygenase* genes was performed with fungal genomes belonging to the Pucciniomycotina. The analysis revealed the incorporation of *TET/JBP* genes in seven lineages of DNA transposons, including *Dileera* (Table S1). Hereafter, DNA transposons encoding a TET/JBP dioxygenase are described as TET^+^. Families of TET^+^ *Dileera* DNA transposons were found from *P. graminis*, *P. triticina* and *Melampsora larici-populina*.

#### 3.1.2. hAT7 in hAT

**Figure 2:**
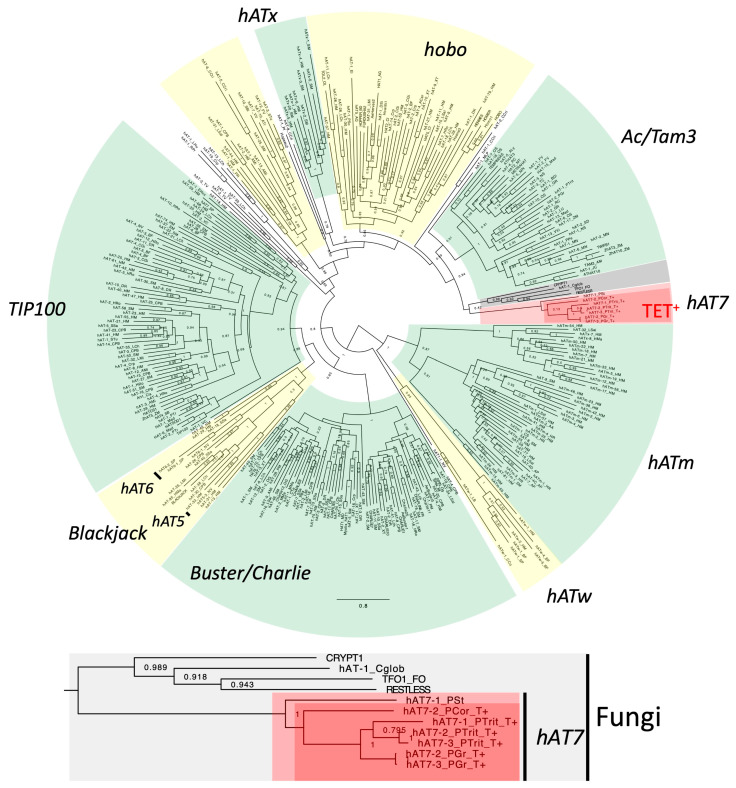
Phylogeny of the *hAT* superfamily of DNA transposons. The maximum-likelihood tree was generated at the PhyML 3.0 server. Approximate likelihood-ratio test for branches (aLRT) values are shown at nodes. The substitution model LG + G + I + F was used. Highlighted in yellow, in green, or in gray are the lineages supported by aLRT values over 0.8. Highlighted in gray is the lineage found from fungi. The lineage of *hAT7* is highlighted in light red, and the lineage of TET^+^ *hAT7* is highlighted in dark red. The subtree for TET^+^ transposons is shown below the entire phylogeny. TET^+^ families are indicated with “T+” after the family names. The phylogenetic tree in the Newick format is available as Data S2.

Twenty families of TET^+^ DNA transposons from six *Puccinia* species (*P. graminis*, *P. triticina*, *P. coronata*, *P. horiana*, *P. hordei*, and *P. novopanici*) were classified as *hAT* based on the similarity of encoded transposases (Table S1), although they generate 7 bp TSDs (Figure 1). While DNA transposons belonging to the *hAT* superfamily usually generate 8 bp TSDs, *hAT* families with 5 bp TSDs and with 6 bp TSDs have been characterized and designated as *hAT5* and *hAT6*, respectively [31,32]. The *hAT* families with 7 bp TSDs characterized here are designated as *hAT7*. *hAT7* families were also found from *P. striiformes* and *M. larici-populina* (Data S1), but these families do not encode a TET/JBP dioxygenase. Phylogenetic analysis confirmed their phylogenetic positions within the *hAT* superfamily (Figure 2). All *hAT7* families clustered with other fungal *hAT* and plant *hAT* families are represented by *Ac* and *Tam3*. *hAT5* and *hAT6* are positioned inside of a lineage including *Blackjack* and other animal *hAT* families.

#### 3.1.3. IS3EU

A set of TET^+^ DNA transposons from seven species of Pucciniales fungi (*P. graminis*, *P. striiformis*, *P. triticina*, *P. coronata*, *P. horiana*, *M. larici-populina*, and *M. medusae*) generate 6 bp TSDs and were classified as *IS3EU* (Figure 1; Table S1). They have TIRs longer than 30 bps and end with TAYGG..CCRTA.

#### 3.1.4. PHISTA in PHIS (PIF/Harbinger-ISL2EU-Spy)

**Figure 3:**
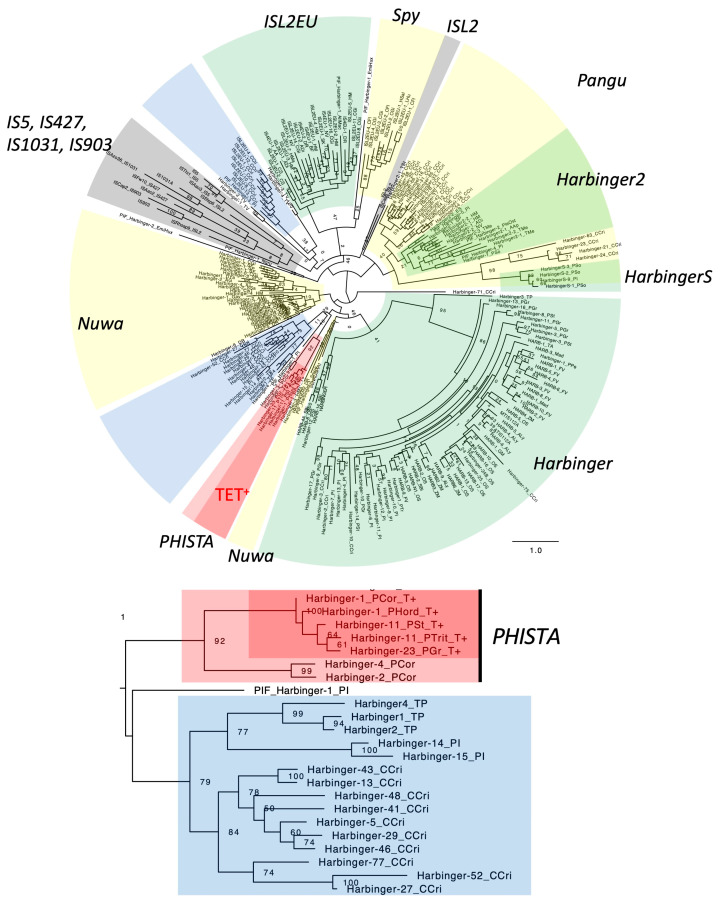
Phylogeny of the *Harbinger and ISL2EU* superfamilies of DNA transposons with prokaryotic IS*5* family of insertion sequences. The maximum-likelihood tree was generated at the PhyML 3.0 server. Bootstrap values of 100 replicates are shown at nodes. The substitution model LG + G + I + F was used. Recognizable groups in the *Harbinger* (*Harbinger*, *Harbinger2*, *HarbingerS*, *Pangu*, *Nuwa*, and *PHISTA*) and *ISL2EU* (*ISL2EU* and *Spy*) superfamilies and in the IS*5* family (IS*5*, IS*427*, IS*1031*, IS*903*, IS*L2*) are highlighted in different colors. Highlighted in light red is the *PHISTA* lineage, and the TET^+^ *PHISTA* lineage is highlighted in dark red. The subtree for TET^+^ transposons is shown below the entire phylogeny. TET^+^ families are indicated with “T+” after the family names. The phylogenetic tree in the Newick format is available as Data S3.

Fifteen families of TET^+^ DNA transposons generating TSDs of TA dinucleotides from six species of Pucciniales fungi (*P. graminis*, *P. striiformis*, *P. triticina*, *P. coronata*, *P. hordei*, and *Austropuccinia psidii*) were classified as *Harbinger* in the classification system of Repbase (Figure 1; Table S1). Two superfamilies, *Harbinger* and *ISL2EU* in Repbase [14], correspond to the *PHIS* (*PIF/Harbinger-ISL2EU-Spy*) superfamily in the definition by Han et al. [33]. Canonical *Harbinger* transposons generate 3 bp TSDs, but DNA transposons related to *Harbinger* generate 0 bp, 2 bp, or 3 bp TSDs [14,33,34]. TET^+^ *Harbinger* families from the genus *Puccinia* are very similar to one another and likely share the common TET^+^ ancestor. To confirm the phylogenetic positions of these transposons, the phylogenetic analysis of DDE transposases was performed (Figure 3). The entire phylogeny is consistent with the one reported in Han et al. [33]. Although the statistical supports are not high, three lineages of eukaryotic transposons can be observed. One is *ISL2EU* and *Spy*; *Spy* is classified as *ISL2EU* in Repbase. *Harbinger2* in Repbase corresponds to *Pangu* in Han et al. [33], and *HarbingerS* is related to *Harbinger2/Pangu*. The last lineage is composed of canonical *Harbinger* families and *Nuwa*.

TET^+^ *Harbinger* families and TET^-^ *Harbinger* families from *Puccinia* clustered together. This lineage was positioned near or inside of the *Nuwa* lineage although the statistical support was weak. Three families (*PIF_Harbinger-1_PI*, *Harbinger4_TP* and *Harbinger-29_CCri*) in the sister lineage of TET^+^ *Harbinger* families were confirmed to generate 3 bp TSDs (Figure S1), consistent with the report that *Nuwa* families generate 3 bp TSDs [33]. *Nuwa* families encode two proteins: the transposase protein and a DNA-binding protein with a Myb/SANT domain. TET^+^ *Harbinger* families encode the third protein in addition to the transposase and the TET/JBP dioxygenase, but none of these third proteins contains a Myb/SANT domain. These differences do not support the classification of the lineage of these families from *Puccinia* to *Nuwa*. Here, the lineage of these families from *Puccinia* is designated as *PHISTA* (*PHIS* transposon with TA TSDs). *PHISTA* belongs to the *Harbinger* superfamily in the classification in Repbase.

#### 3.1.5. EnSpm in CMCT (CACTA/EnSpm-Mirage-Chapaev-Transib)

**Figure 4:**
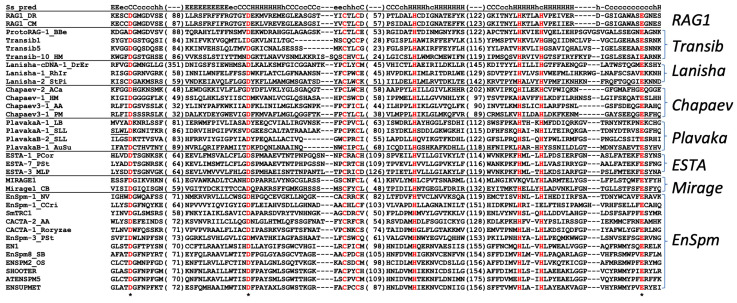
Motifs conserved by the CMCT clade of DDE transposases. Three catalytic residues (D, D, E; asterisks below) and five conserved residues (2C and 3H) are colored in red. The top line Ss_pred indicates the predicted secondary structure of zebrafish RAG1 transposase. Less-conserved segments are omitted and the length of peptides are shown in parentheses.

Three families of TET^+^ DNA transposons generating 3 bp TSDs from *P. striiformis* show the sequence similarity to *Plavaka* transposase proteins. However, the Censor search against Repbase with the predicted protein sequences of these transposons showed a weak similarity with *EnSpm* families instead. The termini 5′-CAC..GTG-3′ and 3 bp TSDs are consistent with the classification as *EnSpm*, and thus, these families are classified as *EnSpm* (Figure 1; Table S1). Related *EnSpm* families were also found from six other species (*P. striiformis*, *P. triticina*, *P. coronata*, *P. horiana*, *P. hordei*, *A. psidii*). No TET^+^ *Plavaka* families were found from Pucciniomycotina in this study. The transposases of *Plavaka* show recognizable similarity to *EnSpm* transposases, and thus, the placement of *Plavaka* related to the *KDZ* group in my previous review [15] should be corrected. The transposases of *Plavaka* contain C(2)C and H(3-4)H motifs between the second D and the last E catalytic residues like *EnSpm* and *Transib* (Figure 4).

#### 3.1.6. ESTA in CMCT (CACTA/EnSpm-Mirage-Chapaev-Transib)

Three TET^+^ DNA transposon families with TSDs of TA dinucleotides do not resemble *PHISTA* families described above. The structure-based sequence comparison with HHpred (https://toolkit.tuebingen.mpg.de/tools/hhpred (accessed on 21 March 2019)) revealed the similarity of predicted transposases with recombination-activating gene 1 (RAG1). It is known that RAG1 originated from the transposase protein of *Transib* DNA transposons [18], and *Transib* shares some sequence motifs with *EnSpm* [17]. These TET^+^ DNA transposon families are designated as *ESTA* (*EnSpm*-like DNA transposons with TA TSDs) here (Figure 1; Table S1).

**Figure 5:**
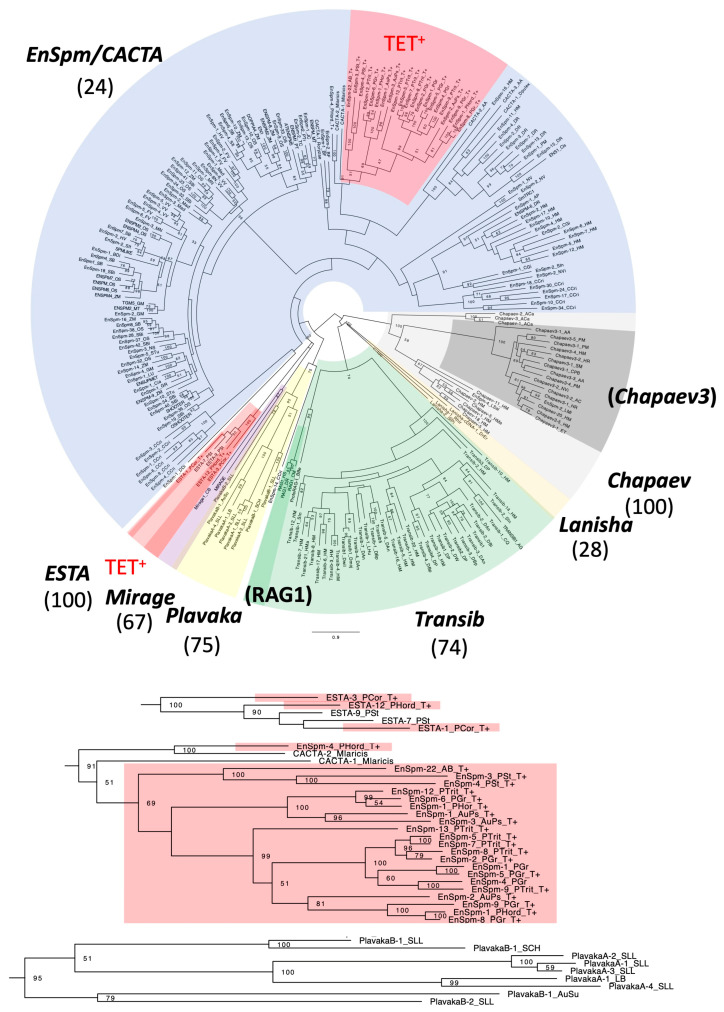
Phylogeny of the CMCT clade of DNA transposons. The maximum-likelihood tree was generated at the PhyML 3.0 server. Bootstrap supports for the lineages of DNA transposons are shown in parentheses. The substitution model LG + G + I + F was used. Recognized lineages are highlighted in different colors. TET^+^ lineages are highlighted in dark red. *Chapaev3* is a sublineage of *Chapaev*. RAG1 originated from *Transib*. Subtrees of *ESTA*, TET^+^ *EnSpm*, and *Plavaka* are shown below the entire phylogeny. TET^+^ families are indicated with “T+” after the family names. The phylogenetic tree in the Newick format is available as Data S4.

The sequence alignment of transposase domains revealed that *EnSpm*, *Mirage*, *Chapaev*, *Transib*, *Plavaka*, *ESTA*, and *Lanisha*, as well as RAG1, all share C(2)C and H(3-4)H motifs between the second D and the last E catalytic residues (Figure 4). *Lanisha* is a recently reported lineage of DNA transposons showing similarity to *EnSpm* [35]. The phylogenetic analysis of transposases indicates the distinction of *Plavaka* and *ESTA* from other transposon lineages (Figure 5). The term CMC was introduced to represent the lineage of *EnSpm* (also called *CACTA*), *Mirage*, and *Chapaev* [17]. Because of the large divergence in sequence, it is not certain whether *Plavaka*, *ESTA*, and *Lanisha* are lineages inside or outside CMC. To avoid uncertainty, the term CMCT clade is here introduced to represent this diverse group of DNA transposons, including *EnSpm*, *Mirage*, *Chapaev*, *Transib*, *Plavaka*, *ESTA*, and *Lanisha*. These DNA transposon lineages share 5′-CA..TG-3′ termini in most cases and generate 2 to 5 bp TSDs. In the CMCT phylogeny, fungal *EnSpm* and *ESTA* are two distinct lineages.

#### 3.1.7. Helitron1 in Helitron

Five families of *Helitrons* found in the genome of *P. graminis* encode a TET/JBP dioxygenase (Figure 1; Table S1). *Helitron* DNA transposons can be classified into two groups: *Helitron1* and *Helitron2* [36]. The TET^+^ *Helitron* families from *P. graminis* belong to *Helitron1*.

### 3.2. DNA Transposons Encoding a TET/JBP Dioxygenase in Agaricomycotina, Basidiomycota

#### 3.2.1. Kyakuja in KDZ (Kyakuja-Dileera-Zisupton)

Two families of TET^+^ DNA transposons (*Kyakuja-1_LB and Kyakuja-2_LB*) were characterized in this analysis using protein sequences of *Kyakuja* from *Laccaria bicolor* [21]. They generate 7 bp TSDs (Figure 1). They show 5′-CA..TG-3′ termini but have no TIRs. Using these TET/JBP protein sequences as queries for homology search, systematic screening of TET^+^ DNA transposons was performed from fungal genomes belonging to the Agaricomycotina. The analysis revealed the incorporation of *TET/JBP* genes in five lineages of DNA transposons, including *Kyakuja* (Figure 1; Table S1). In contrast to Pucciniomycotina, where many different families of TET^+^ DNA transposons were found from each genome, in Agaricomycotina, most genomes encode a few TET^+^ DNA transposon families. The genomes of five orders of Agaricomycotina fungi contain TET^+^ DNA transposons. *Kyakuja* was found from all of these orders. From Auriculariales, TET^+^ *Zisupton* DNA transposons were characterized in addition to *Kyakuja*. From Agaricales, four lineages (*Zisupton*, *EnSpm*, *Plavaka*, and *Helitron2*) of TET^+^ DNA transposons were characterized from *Agaricus bisporus*. The other three orders (Boletales, Polyporales, and Russulales) show the presence of only TET^+^ *Kyakuja* DNA transposons.

**Figure 6:**
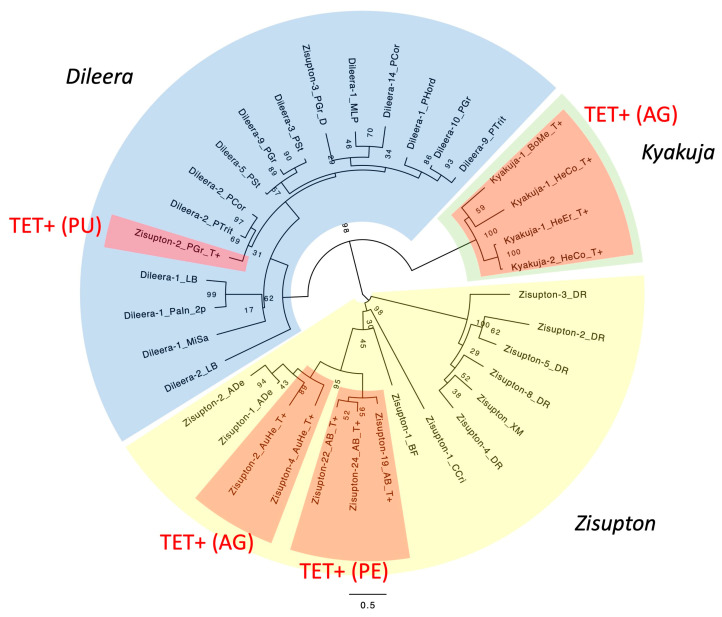
Phylogeny of the *KDZ* lineage of DNA transposons. The maximum-likelihood tree was generated at the PhyML 3.0 server. Bootstrap supports of 100 duplicates are shown at nodes. The substitution model WAG + G + F was used. *Zisupton*, *Dileera*, and *Kyakuja* are highlighted in different colors. TET^+^ lineages are highlighted in dark red, and the type of TET/JBP dioxygenases is indicated in parentheses.

TET^+^ *Kyakuja* families were found from diverse fungal species in Agaricomycotina (Figure 1; Table S1). While *Zisupton* and *Kyakuja* show some similarity between their transposase proteins, in the phylogeny of transposases, *Zisupton*, *Dileera*, and *Kyakuja* are well separated (Figure 6). TET^+^ *Zisupton* families found from Auriculariales and Agaricales show a clear distinction from TET^+^ *Kyakuja* families. It is also obvious that the acquisition of *TET/JBP dioxygenase* gene in these three lineages (*Kyakuja*, *Zisupton* in Auriculariales, and *Zisupton* in Agaricales) was independent from that in *Dileera* in Pucciniales.

#### 3.2.2. Plavaka in CMCT (CACTA/EnSpm-Mirage-Chapaev-Transib)

*Plavaka* families with or without *TET/JBP* genes were reconstructed from various fungi in Agaricomycotina (Data S1). *Plavaka* families from two species of Agaricales fungi, *A. bisporus* and *Gymnopus confluens*, encode a TET/JBP dioxygenase (Figure 1; Table S1). The association between *Plavaka* and *TET/JBP* genes were implied in *P. graminis*, *A. bisporus*, *Serpula lacrymans* (Boletales), *Fomitopsis pinicola* (Polyporales), and *Trametes versicolor* (Polyporales) [21]. TET^+^ *Plavaka* families were not found from any of these fungi except *A. bisporus*, although six TET^-^ *Plavaka* families were reconstructed from the genome of *S. lacrymans* (Figure S2).

Phylogenetic analysis of transposase domains revealed two distinct lineages inside of *Plavaka* (*PlavakaA* and *PlavakaB*) (Figure 5). This classification is consistent with their terminal features (Figure S2). *PlavakaA* families generate 2 bp TSDs and their termini are 5′-TGT..ACA-3′. *PlavakaB* families generate 2 or 3 bp TSDs and their termini are 5′-CATCA..TGATG-3′. TET^+^ *Plavaka* families all belong to *PlavakaB*.

#### 3.2.3. Distinct TET/JBP Dioxygenase-like Proteins

During the analysis of transposons from *Agaricus bisporus*, several proteins similar to TET/JBP dioxygenases were recognized. These proteins are very distantly related to known TET/JBP dioxygenases, and the similarity was indicated through structure-based alignment with HHpred (https://toolkit.tuebingen.mpg.de/tools/hhpred (accessed on 19 November 2025)) with the e-value 1.4 for the *Chlamydomonas reinhardtii* TET homolog CMD1, and 19 for *Naegleria* Tet-like dioxygenase NgTET. AlphaFold Protein Structure Database (https://www.alphafold.com/ (accessed on 19 November 2025)) includes many predicted structures of these TET/JBP dioxygenases, such as K5WYP2, A0A8H7KFD6, and A0A8H7C4L8, and they show significant similarity to the resolved structures of CMD1, NgTET, and a TET dioxygenase from the fungus *Coprinopsis cinerea* (CcTET) proteins (e-values <1 × 10^−5^). These proteins are designated as PE-type TET/JBP dioxygenases and the detailed analysis is described in the chapter 3.4. This group of TET/JBP dioxygenases were found from the transposons of *A. bisporus*, *Laccaria bicolor*, and *Gymnopus confluens*.

### 3.3. DNA Transposons Encoding a TET/JBP Dioxygenase in Pezizomycotina, Ascomycota

Using the TET/JBP protein sequences from *L. bicolor* as queries for homology search, systematic screening of TET^+^ DNA transposons was performed from fungal genomes belonging to Ascomycota. Two lineages of DNA transposons (*Helitron* and *IS3EU*) were revealed to encode a TET/JBP dioxygenase (Figure 1; Table S1). A TET^+^ *IS3EU* family was found only from *Tuber indicum*, while TET^+^ *Helitron* families were found from five species from four fungal families (Ascobolaceae, Tuberaceae, Morchellaceae, and Pyronemataceae) in the order Pezizales. These TET^+^ *Helitrons* belong to *Helitron1*.

### 3.4. Phylogenetic Relationships of TET/JBP Dioxygenases and Possible Mechanisms of Horizontal Transmission

**Figure 7:**
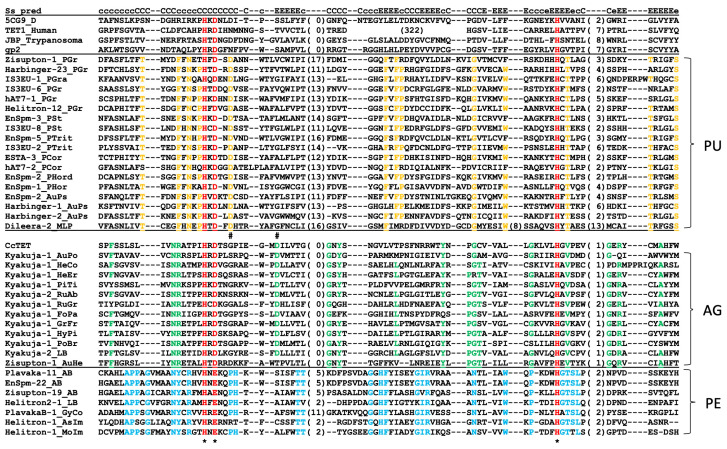
Alignment of TET/JBP dioxygenase domains encoded by TEs. The top line Ss_pred indicates the secondary structure of *Naegleria gruberi* NgTET1 (5CG9_D). Cc: alpha helix, Ee: beta-sheet. Conserved residues among all three lineages (H, D/E, and H; asterisks below) are colored in red, while conserved residues in each lineage are colored in yellow (PU), green (AG), or cyan (PE). The conserved residues among all TET/JBP dioxygenases are indicated with *, and the residues related to the unique function of CcTET are indicated with #. Less-conserved segments are omitted and the length of peptides are shown in parentheses. TET1_Human: human methylcytosine dioxygenase TET1; JBP_Trypanosoma: *Trypanosoma brucei* j-binding protein; gp2: gp2 from *Mycobacterium* phage Cooper; CcTET: a TET dioxygenase from *Coprinopsis cinerea* (A8P1J0). Species abbreviations in TE family names are shown in Table S1.

TET/JBP dioxygenase proteins encoded by transposons can be divided into three groups based on the conserved residues (Figure 7). These three groups mostly correspond to their host classification: Pucciniomycetes, Agaricomycetes, and Pezizomycetes with some exceptions. Thus, they are designated as PU, AG, and PE types, respectively. The PE-type *TET/JBP dioxygenase* genes are encoded by DNA transposons from Agaricales in Agaricomycetes as well as from Pezizomycetes. They are too diverged to reliably analyze phylogenetic relationships, and therefore, PU, AG, and PE types of TET/JBP dioxygenases were analyzed separately.

The structure of CcTET, a TET dioxygenase encoded in the genome of *Coprinopsis cinerea*, was reported [37,38]. The sequence comparison clarified that the CcTET is a close relative to the AG-type TET/JBP dioxygenases encoded by *Kyakuja* and *Zisupton* DNA transposons (Figure 7). CcTET converts 5mC to 5hmC, 5-formylcytosine, and 5-carboxylcytosine, as does its mammalian homologs [39]. CcTET also oxydizes N^6^-methyladenosine (6mA) to N^6^-hydroxymethyladenosine (6hmA) in duplex DNA [37,38]. Two residues Gly331 and Asp337 (Figure 7, indicated by #), just downstream from the conserved residues His326 and Asp328 for ion binding, facilitated 6mA recognition and catalysis. The Asp337 is essential for the catalytic activity on 5mC, while not essential for 6mA demethylation. The Asp337 has a central role in compensating for the loss of a critical 5mC-stablizing H-bond observed in conventional TET enzymes, and stabilizes 5mC and subsequent intermediates through an H-bond with the N4 atom of the substrates. The orthologous position of Asp337 is conserved among the AG-type TET dioxygenases encoded by *Kyakuja* transposons (Figure 7). At the corresponding position of Gly331, Gly or Ser are observed among the *Kyakuja* transposons. The conservation at both positions strongly suggests that the AG-type TET also has the potential to catalyze both 5mC and 6mA.

In the PE-type TET dioxygenases, HxE is conserved instead of the HxD motif in other TET/JBP dioxygenases. This substitution is a hallmark of the PE type. The HxD/E and the conserved downstream H residue (Figure 7, asterisks) constitute the 2-His-1-carboxylate facial triad, which bind Fe(II) in the wide Mononuclear non-heme Fe(II)-and 2-oxoglutarate (2OG)-dependent oxygenase superfamily [40]. Thus, the motif HxE is likely to function similar to the HxD motif in other dioxygenases.

The structure-based homology analysis with HHpred (https://toolkit.tuebingen.mpg.de/tools/hhpred (accessed on 19 November 2025)) and at AlphaFold Protein Structure Database *(*https://www.alphafold.com/ (accessed on 19 November 2025)) indicated that the most closely related proteins whose structure have been characterized to the PU-type and the PE-type TET/JBP dioxygenases were CMD1, CcTET, NgTET, and human TET2. CcTET, NgTET, and human TET2 show the dioxygenase activity against 5mC, 5hmC, and 5-formylcytosine. CMD1 shows a distinct activity; it catalyzes the conjugation of a glyceryl moiety to the C^5^-methyl group of 5mC, leading to C^5^-glyceryl-methylcytosine (5gmC) [41]. It is likely that the PU- and PE-type TET/JBP dioxygenases have the same catalytic activity as TET, but another activity, such as the modification on cytosine or thymine on DNA, cannot be excluded.

**Figure 8:**
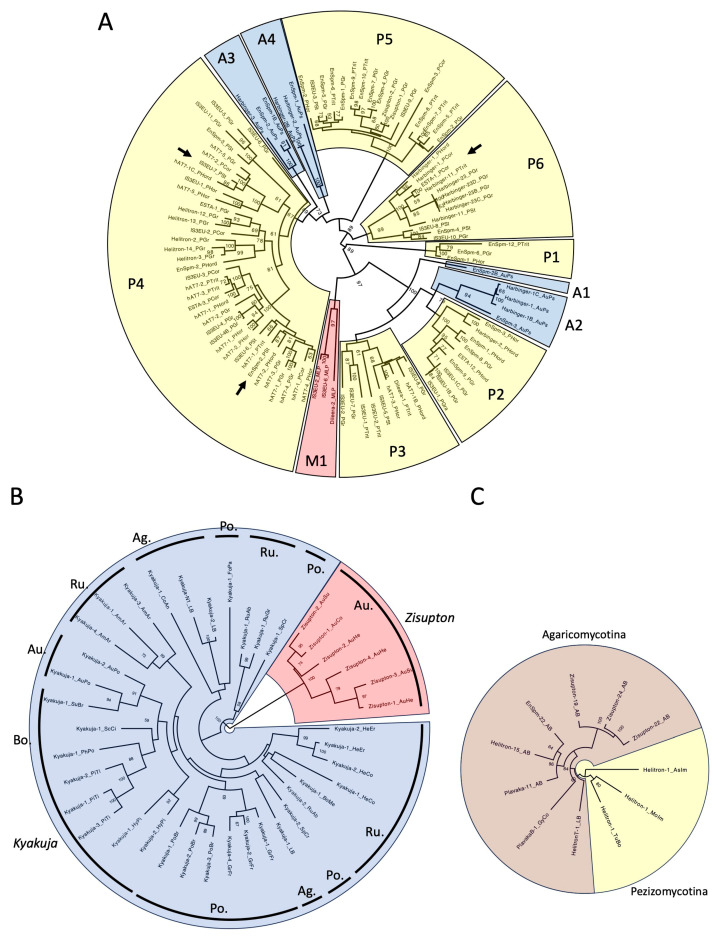
Phylogeny of TET/JBP dioxygenase domains encoded by TEs. Maximum-likelihood trees were generated at the PhyML 3.0 server. Bootstrap supports over 50% are indicated at nodes. The substitution model LG + G + I + F was used in the phylogenetic analyses of PU- and AG-type, and LG + G + I was used in the phylogenetic analysis of PE-type. Species abbreviations in TE family names are shown in Table S1. (**A**). PU-type. The clusters of TEs from the same genus are numbered and colored in yellow (*Puccinia*), in blue (*Austropuccinia*) or in red (*Melampsola*). Arrows indicate the possible horizontal transfer events between DNA transposon superfamilies (Figure 9). (**B**). AG-type. Fungal families from which DNA transposons are characterized are indicated: Au., Auriculariales; Ag., Agricales; Bo., Boleales; Po., Polyporales; and Ru., Russulales. (**C**). PE-type.

Phylogenetic analysis of TET/JBP dioxygenase genes incorporated in different DNA transposons was performed (Figure 8).

**Figure 9:**
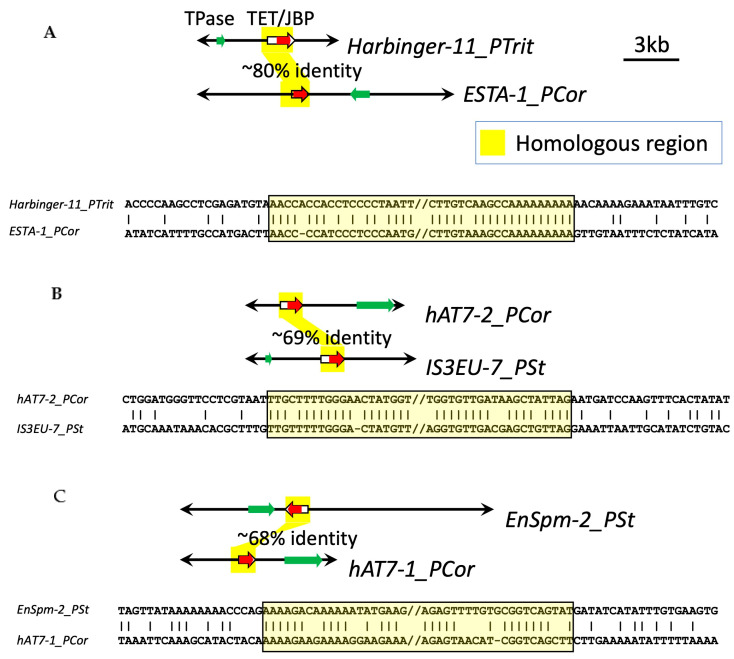
Schematic comparisons of *TET/JBP* gene sequences in TET^+^ DNA transposons. Arrows indicate the entire transposon sequences with TIRs. Homologous regions are highlighted in yellow. (**A**) *Harbinger-11_PTrit* and *ESTA-1_PCor*, (**B**) *hAT7-2_PCor* and *IS3EU-7_PSt*, (**C**) *EnSpm-2_PSt* and *hAT7-1_PCor*.

In the PU-type, TET/JBP genes are clustered independently from the superfamilies of DNA transposons, but dependently on the host genera (Figure 8A). Six clusters for *Puccinia* (P1 to P6), four clusters for *Austropuccinia* (A1 to A4), and one cluster for *Melampsola* (M1) were observed. Well-supported clusters include different superfamilies of DNA transposons. For examples, the P2 cluster includes *EnSpm*, *PHISTA*, *ESTA*, and *IS3EU* and the P3 cluster includes *IS3EU*, *hAT7*, and *Dileera*. Such clustering indicates frequent transfer of *TET/JBP* genes between superfamilies of DNA transposons.

Although it is, in general, difficult to determine the direction of *TET/JBP* gene transfer between transposons, the direction is implied in some cases. There is only one case where the nucleotide sequences for TET/JBP dioxygenases from different DNA transposons show over 70% sequence identity. It is the transfer to *ESTA-1_PCor* from *PHISTA* families (Figure 8A, the arrow in P6). TET^+^ *PHISTA* families, in the P6 cluster, are observed in different species of *Puccinia* (*P. graminis*, *P. striiformes*, *P. triticina*, *P. coronata*, and *P. hordei*). These families are considered to be orthologous, based on the sequence similarity and gene composition. *TET/JBP* gene sequences from *Harbinger-11_PTrit* and *ESTA-1_PCor* are ~80% identical to each other (Figure 9). Both ends of homologous sequences do not have specific structure or sequence. There are no inverted repeats, no direct repeats, or no palindromic sequence. The comparison between *hAT7-2_PCor* and *IS3EU-7_PSt*, and between *EnSpm-2_PSt* and *hAT7-1_PCor*, both in the P4 cluster (Figure 8A, the arrows in P4) showed that their *TET/JBP* genes are less than 70% identical to each other (Figure 9). In those cases, there is no signature sequence at either end of homologous sequences.

In contrast to the mixed superfamilies in the PU-type, the AG-type shows no horizontal transmission between transposon superfamilies. *TET/JBP* genes from *Kyakuja* and those from *Zisupton* were clearly separated (Figure 8B). *TET/JBP* genes from the same fungal family tend to cluster together. Due to the low resolution among *Kyakuja* families, horizontal transfer between species is not supported.

In the PE-type, which contains the lowest number of TET/JBP sequences, sequences from Pezizomycotina appear more diverged than those from Agaricomycotina, suggesting their origin in Pezizomycotina (Figure 8C). *IS3EU-1_TuIn* and *Helitron-1_SpBr* were removed from the analysis as they have a large deletion at the *N*-terminal region of TET/JBP domain. The wider distribution of the PE type in Pezizomycotina (five families in one order) than in Agaricomycotina (one family in one order) also support their origin in Pezizomycotina. TET/JBP protein sequences from *A. bisporus* encoded by different transposon superfamilies clustered together, suggesting the horizontal transfer of *TET/JBP* genes between superfamilies.

## 4. Discussion

### 4.1. The Mode of Transmission of TET/JBP Genes Between Superfamilies of Transposons

In this study, it is shown that different superfamilies of DNA transposons in fungi encode related TET/JBP dioxygenases. These *TET/JBP* genes are transferred between superfamilies in Pucciniomycotina and likely also in Agaricomycotina.

It is unlikely that the *TET/JBP* genes themselves are non-autonomous transposons. The comparison of related *TET/JBP* genes in different transposon families show no terminal motifs or structures supporting their own transposition (Figure 9). No TSDs were recognized either. *TET/JBP* genes seem passively transferred across transposon families.

The conservation of gene order and orientation is observed among some transposon families. All TET^+^ *IS3EU* families contain a *TET/JBP* gene between their transposase and alpha-kinase genes. Transposase and alpha-kinase genes are in the opposite orientations. In three *IS3EU* families, *IS3EU-7_PSt*, *IS3EU-3_PCor*, and *IS3EU-6_PSt*, the *TET/JBP* gene is in the same orientation as transposase. They belong to the same TET/JBP lineage (P4), although their relatives also include many different superfamilies of transposons (Figure 8). *hAT7-1_PCor*, *hAT7-2_PCor* and *hAT7-1_PHor*, which also belong to the TET/JBP lineage P4, encoding a *TET/JBP* gene upstream of their transposase in the same orientation. Although the same gene order and orientation may reflect their shared ancestry, the possibility of parallel captures of *TET/JBP* genes at similar locations inside of transposons cannot be ruled out. The evolutionary constraint on the regulation of expression, or the positions of non-essential sequences may lead to parallel evolution.

### 4.2. TET/JBP Dioxygenases and Fungal Transposon-Silencing Mechanisms

The function of the *TET/JBP* genes encoded by fungal DNA transposons is not experimentally validated, but given that their protein-coding regions are mostly not disrupted, their functionality in the lifecycle of DNA transposons seems to have been conserved. The presence of multiple TET^+^ transposon families in the same genome, especially in Pucciniomycotina, strongly suggests that each TET/JBP dioxygenase contributes to the fitness of its own transposon family. It is unlikely that these *TET/JBP dioxygenase* genes primarily work for the fitness of the host organism.

It is noteworthy that no retrotransposon was found to encode a TET/JBP dioxygenase. Many LTR retrotransposons have been characterized from fungi, including Pucciniomycotina [14]. Most of TET^+^ transposons are DNA transposons which encode a DDD/E transposase, while some *Helitrons* also encode a TET/JBP dioxygenase. *Helitrons* encode an HUH nuclease/Y1 transposase for single-stranded transposition, distinct from the double-stranded transposition seen in DNA transposons with a DDD/E transposase.

As DNA methylation regulates the gene expression of transposons [7], it is reasonable to speculate that the TET/JBP dioxygenases encoded by fungal transposons remove the epigenetic silencing marks on the transposon sequences. Metazoan TET proteins oxidize 5mC to 5hmC. TET proteins also catalyze the oxidation of 5hmC to generate 5-formilcytosine, and the oxidation of 5-formilcytosine to generate 5-carboxycytosine. 5-Formilcytosine and 5-carboxycytosine can be removed by thymine DNA glycosylase, leading to the pathway of base excision repair. These oxidized cytosine derivatives are finally replaced by unmodified cytosine.

In fungi, repeat-induced point mutation (RIP) and its related process are the main driver to disrupt the codability of transposons [42]. The RIP process of *Neurospora crassa* causes C-to-T transitions in CA dinucleotides in repeats [43]. RIP requires the RIP defective (RID) C5-DNA-methyltransferase of the Dnmt1 family. The deamination of 5mC results in uracil and the uracil is replaced by thymine during replication. Another fungal defense mechanism against transposons is called methylation, induced premeiotically (MIP), and is also involved in a Dnmt1 cytosine methyltransferase called methyltransferase from *Ascobolus* 1 (Masc1). In MIP, the silenced phenotype can be reversible through the loss of cytosine methylation. It is now known that both RIP and MIP represent two appearances of the same process.

Although RIP is considered to predate the divergence of Dikarya, there is no evidence of RIP in the genomes of Saccharomycotina and Taphrinomycotina, indicating the secondary loss of RIP process in these lineages [44]. In contrast, RIP is widely observed in Pezizomycotina, including the genera *Neurospora*, *Fusarium*, and *Aspergillus*. Masc1/RID homologs were identified in nearly all fungi in Pezizomycotina. The level of activity of RIP is different among species; *Pyrenosphora* and *Neurospora* show the highest activity, while *Fusarium* and *Blumeria* show low RIP activity. Pezizomycetes, represented by the genus *Tuber*, show moderately low RIP activities among Pezizomycotina.

While the RIP activity in *T. melanosporum* is moderately low, this fungus has one of the most highly methylated genome [45]. DNA methylation targets transposons in a nearly exclusive manner. Its genome size is relatively large (125 Mb) among fungi, and transposon contents are high (>58%). There is negative correlation between transposon expression and transposon methylation levels. Therefore, DNA methylation is considered to suppress the expression of transposons in *T. melanosporum*. The counteraction against DNA methylation to escape transcription suppression is likely also the function of TET/JBP dioxygenases encoded by transposons in these fungi without strong RIP-like process.

Among Basidiomycetes, fungi belonging to Pucciniomycotina, including the genera *Puccinia* and *Microbotryum*, show the patterns of hypermutations at TCG trinucleotide sites in repetitive sequences [46]. Instead of RID/Masc1 homologs, these species encode another Dnmt1 gene, called Masc2 [47]. The difference of major Dnmt1 protein in the process likely leads to the different hypermutation patterns between Pezizomycotina and Pucciniomycotina. The very low overall GC content of the genome of *A. psidii*, which is dominated by the *Gypsy* superfamily of LTR retrotransposons, also suggests the hypermutations at CG sites in *Gypsy* LTR retrotransposons [48]. Considering the abundance of TET^+^ DNA transposons and frequent transfer among transposons in *Puccinia*, it is reasonable to speculate that the TET/JBP dioxygenases encoded by transposons counteract the activity of C-to-T hypermutations in transposons.

*Coprinopsis cinerea* is a member of Agaricales, Agaricomycotina. CcTET from *C. cinerea* catalyzes the oxidation of 5mC on DNA to generate 5hmC, 5-formilcytosine, and 5-carboxycytosine [39]. CcTET is a close relative to the AG-type TET/JBP dioxygenases, and likely originated from, or is still a part of, a *Kyakuja* DNA transposon. CcTET also exhibits the catalytic activity on 6mA, unlike other TET dioxygenases [37,38]. Two catalytically important residues for the recognition of 6mA were identified and both residues are basically conserved among AG-type TET/JBP dioxygenases encoded by *Kyakuja* DNA transposons (Figure 7). It is likely that the AG-type TET/JBP dioxygenases encoded by *Kyakuja* also have the dioxygenase activity on 6mA. Given the fact that RIP impacts only on cytosine, the functional significance of the dioxygenase activity on 6mA remains to be elucidated.

In Agaricomycotina, the relationships between the RIP-like process and the function of TET/JBP dioxygenases are not so obvious. *Agaricus bisporus* shows multiple superfamilies of TET^+^ DNA transposons. The close relationship of *TET/JBP* genes in *A. bisporus* indicates the transfer of *TET/JBP* genes between DNA transposons (Figure 5). Hypermutation is not detected among Agaricomycotina [46]. There is no indication of the presence of RIP-like process in *A. bisporus*; however, the CG methylation is almost completely restricted to transposons [49].

The genome of *C. cinerea* contains 46 AG-type *TET/JBP* genes associated with *Kyakuja* DNA transposons, and 32 are likely catalytically active [50]. A genome-wide oxidized methylcytosine survey revealed that the regions surrounding *Kyakuja* transposons, as well as other transposons and centromere, are heavily modified with oxidized forms of methylcytosine (oxi-mC). oxi-mC is mainly colocalized with 5mC. It is noteworthy that retrotransposons were also modified with oxi-mC. *Laccaria bicolor* also shows accumulation of oxi-mC in or near DNA transposons and retrotransposons [51]. It can be speculated that the AG-type TET/JBP dioxygenases in Agaricomycotina oxidize 5mC (and methyladenines) in a non-target specific manner.

Methylation can suppress both DNA transposons and retrotransposons. One possible reason for the lack of TET^+^ retrotransposons is the relatively high copy number of retrotransposons compared with DNA transposons. The activity of TET/JBP dioxygenases may not be sufficient to counteract the activity of cytosine methyltransferases in the RIP-like transposon suppression system.

The counteracting against epigenetic silencing does not have to be the recovery of cytosine. In mammals, 5hmC is not just an intermediate of DNA demethylation, but is an active regulator of transcription and genome stability [52]. 5hmC is linked to active gene regulation, in contrast to stable gene silencing associated with 5mC. There is a possibility that the TET/JBP dioxygenases encoded by transposons would contribute to the transcription control through changing epigenetic marks from 5mC to 5hmC, 5-formilcytosine, and 5-carboxycytosine, as suggested in Chavez et al. [50].

Evidence has also been accumulated that not a few TET/JBP dioxygenases have a role in the hypermodification of pyrimidines on DNA, rather than in the removal of 5mC. The TET/JBP dioxygenases are considered to originate in the arms-race between bacteria and bacteriophages [53]. Phages often encode C5-methyltransferase, TET/JBP dioxygenase, and a couple of glycosyltransferases, and these proteins collaborate to generate a glycosylated cytosine. These modified cytosines are resistant to the attack by restriction endonucleases. The base modification would have another function in eukaryotes, where restriction-modification system is uncommon. CMD1 from *Chlamydomonas reinhardtii* catalyzes the conversion from 5mC to 5gmC using vitamin C as co-substrate [41]. JBP in some kinetoplastids catalyzes the hydroxylation of thymine, which is further glycosylated to become base J [12]. Base J is essential for the proper termination of RNA polymerase II transcription in *Leishmania* [54]. Although transposons with *TET/JBP* genes do not encode additional protein for further modification, such as glycosyltransferases, given the sequence divergence of the PU- and PE-type TET/JBP dioxygenases from the characterized TET dioxygenases, their functions may not be restricted to those of canonical TET dioxygenases. The function of TET/JBP dioxygenases encoded by transposons remains to be elucidated for the better understanding of the fungal epigenetic modification diversity and its biological impacts.

## 5. Conclusions

Multiple lineages of fungal DNA transposons encode a TET/JBP dioxygenase, the only enzyme group that actively catalyzes methylcytosine towards its elimination from DNA. The passive transfer of *TET/JBP dioxygenase* genes between different DNA transposons is indicated through phylogenetic analysis. These transposon-encoded TET/JBP dioxygenases likely contribute to the escape of transposons from the methylation-based silencing system in fungi.

## Data Availability

The original contributions presented in this study are included in the article/Supplementary Materials. Further inquiries can be directed to the corresponding author.

## Supplementary Materials

The following supporting information can be downloaded at: https://www.mdpi.com/article/10.3390/biology14121741/s1, Figure S1: Termini and TSDs of *Harbinger* transposons closely related to TET^+^ lineage; Figure S2: Termini and TSDs of newly characterized *Plavaka* families; Table S1: Fungal DNA transposons with *TET/JBP dioxygenase* genes; Data S1: Sequences of *ESTA*, *Plavaka*, and *hAT7* DNA transposons which do not encode a TET/JBP dioxygenase in fasta format; Data S2: Phylogeny of the *hAT* superfamily of DNA transposons in the Newick format; Data S3: Phylogeny of the *Harbinger and ISL2EU* superfamilies of DNA transposons with prokaryotic IS*5* family of insertion sequences in the Newick format; Data S4: Phylogeny of the CMCT clade of DNA transposons in the Newick format.

## Abbreviations

The following abbreviations are used in this manuscript:

TIR	Terminal inverted repeat
TSD	Target site duplication
aLRT	approximate likelihood-ratio test for branches
RIP	Repeat-induced point mutation
MIP	Methylation induced premeiotically
