# Supplementary material for: Heterogeneous Lineages of DNA Transposons Encode a TET/JBP Dioxygenase in Fungi
Source: Biology (Basel). 2025 Dec 4;14(12):1741. doi: 10.3390/biology14121741 (PMC12730594; doi:10.3390/biology14121741)
Supplement: Supplementary file 1 [file biology-14-01741-s001.zip › FigS.pdf]

PIF_Harbinger-1_PI (Phytophthora infestans T30-4)

```
NW_003303754.1_[2408236-2407419]
CCCATTCTTAGGGGCTGGTCGCGCTGGCTAATGCTGGTTCTGGGTCCGGTGCTTAAGTTA//CTGATACCTACCCCTAGCGAGAACCAGCACCAGCCAGCGCGACCAGCCCCTTACTGTATC
```

Harbinger4_TP (Thalassiosira pseudonana)

```
NC_012069.1_[5789-9557]
ATTCTAGTTAGGG--TGTCCAAACCAAAGAAAAAAATCATAGTAGTCATATAATAATCAT//ACTACTATTATATGATTTATGTGATTTTTTTCTTTGGTTTGGACACTCCCTTAGGGTCTC
NC_012076.1_[515189-518959]
TATCCCCTAAGGGGGGGTCCAAACCAAAGAAAAAAATCATAGTAGTCATATAATAATCAT//ACTACTATTATATGATTTATGTGATTTTTTTCTTTGGTTTGGACACTCCCTAAATGATGA
NC_012078.1_[20073-23784]
ACGTGAGTGAGGGCTTGTCCAAACCAATGAAAAAAATCATAGTAATCATA----------//ATGATTATTATATGACTACTATGATTTTTTTCATTGGTTTGGACAAGCCCTGAGGGAGTG
```

Harbinger-29_CCri (Chondrus crispus)

```
NW_005178983.1_[201684-199659]
GGAACCTTTGAGGGAGGGTCTTAACTTGCCCGAGTTTGCTTTGCTTGCCCGAGTTTGCCG//GCACTCGCTCCTTCTGCAGATGAAATTAGTGACACTGGAGTAGCAAGCCCTTGTCTGGCC
NW_005179034.1_[99582-97555]
CTCTCATGTGAGGGAGGGTCTTAACTTGCCCGAGTTTGCTTTGCTTGCCCGAGTTTGCCG//GCACTCGCTCCTTCTGCAGATGAAATTAGTGACACTGGAGTAGCAAGCCCGTGTCTGGCC
```

Figure S1. Termini and target site duplications (TSDs) of *Harbinger* transposons closely related to TET^{+} lineage. TSDs are shown in red. The accession numbers and positions of *Harbinger* insertions are indicated.

PlavakaA-1_LB (Laccaria bicolor)

DS547106.1_[35286-27467]
TAAATACCTATGTCGGAAAAATCCGTGATGCCTTACAATAATAATAAATTACAAGGCTAT//GTGATTACGTGCCTTACCTGCCTTATAAGGCTTCCCGGGTTTTTCCGACATAACATTCTA
DS547110.1_[677717-670427]
AATAGATCTATGTCGGAAAAATCCGTGATGCCTTACAATAATAATAAATTACAAGTCTAT//GTGGTTACGTGCCTTACCTGCCTTATAAGGCTTCCCGGGTTTTTCTGACATAGCTTCACA
DS547121.1_[517275-526642]
TAGCCTCAGATGTCGGAAAAATCCGTGATGCCTTACAATAATAATAAATTACAAGGCTAT//GTGATTACGTGCCTTACCTGCCTTATAAGGCTTCCCGGGTTTTTCCGACAGAGGCCGCTT
DS547146.1_[67201-49666]
GGACGTCCAATGTCGGAAAAATCTGTGATGCCTTACAATAATAATAAATTACAAGGCTAT//GTGATTACGTGCCTTACCTGCCTTATAAGGCTTCCCGGGTTTTTCCGACAAAGTTTGTGG
DS547160.1_[60860-70614]
AGGACTGCTGTGTCGGAAAAATCCGTGATGCCTTACAATAATAATAAATTACAAGGCTAC//GTGATTACGTGCCTTACCTGCCTTATAAGGCTTCCCGGGTTTTTCCGACATGTTGGCGTC
DS547168.1_[40997-49326]
AGCCCTTGTTTGTCAGAAAAATCCGTGATGCCTTACAATAATAATAAATTACAAGGCTAT//GTGATTACGTGCCTTACCTGCCTTATAAGGCTTCCCGGGTTTTTCCGACATTGACTTGGG

PlavakaA-1_SLL (Serpula lacrymans)

GL945428.1_[5302458-5294322]
ATCAACCCGTTGTCGCAAAAATCCGTTTTTCATGATTTGGCCGAATGCGACCTGATAACG//AGTTACAGACATTCGTAGGGTCTGATCTGGTTAAACGGATTTTTCCGACAGTTGTCCCAC
GL945430.1_[2151686-2159821]
GCCCAGCGCATGTCGCAAAAATCCGTTTTTCATGATTTGGCCGAATGCGACCTGATAACG//AGTTACAGACATTCGTAGGGTCTGATCTGGTTAAACGGATTTTTCCGACACAGTTCGCAG
GL945432.1_[1476560-1484695]
TCAGAAACTCTGTCGCAAAAATCCGTTTTTCATGATTTGGCCGAATGCGACCTGATAACG//AGTTACAGACATTCGTAGGGTCTGATCTGGTTAAACGGATTTTTCCGACATCTCTTCCTT
GL945433.1_[746525-738390]
GAAGTAGGTTTGTCGCAAAAATCCGTTTTTCATGATTTGGCCGAATGCGACCTGATAACG//AGTTACAGACATTCGTAGGGTCTGATCTGGTTAAACGGATTTTTCCGACATTTAGACTCA

PlavakaA-2_SLL (Serpula lacrymans)

GL945429.1_[1570020-1561530]
GAAGATCATATGTCGGAAAAATCCATTTTCCCATACAAGGTCTAGAATGGCCACTTCAGG//TCGCAGCATGCTCCAGCAGTCCTGATCAGGTGAATTGGATTTTTCCGACATAGACATCAT
GL945443.1_[362663-354153]
TACGAAATCATGTCGGAAAAATCCATTTTCCCATACAAGGTCTAGAATGGCCACTTCAGG//TCGCAGCATGCTCCAGCAGTCCTGATCAGGTGAATTGGATTTTTCCGACACAAGATTCAT

PlavakaA-3_SLL (Serpula lacrymans)

GL945433.1_[1360414-1352154]
AAAGGTGCGTTGTCGGAAAAATCCACTTTCCATGGTTAGGCCGAGTGTGGTCTGATAAGA//TATCTCAGATACTCACAGTGTCTGATCTGCTAAAATGGATTTTTCCGACAGTGGCTCGAT

PlavakaA-4_SLL (Serpula lacrymans)

GL945428.1_[3266498-3257428]
ATGGAGCATATGTCGGAGATTTCCTATACTGGTTGCAAGAGCACGTGATTGGGTTGGTGT//GCTTGCAATCCAGTGCAATACCTTGCAGCCACTTCCAGAAATCTCTGACATAGCCACCAC
GL945435.1_[608313-599215]
ACAGGCACAGTGTCGGAGATTTCCTATACTGGTTGCAAGAGCACGTGATTGGGTTGGTGT//GCTTGCAATCCAGTGCAATACCTTGCAGCCACTTCCAGAAATCTCCGACAAGTGCCACGT
GL945446.1_[244465-235365]
GCACGAGCAATGTCGGAGATTTCCTATACTGGTTGCAAGAGCACGTGATTGGGTTGGTGT//GCTTGCAATCCAGTGCAATACCTTGCAGCCACTTCCAGAAATCTCCGACAAAGCCCCAAC
GL945446.1_[331576-340672]
AAGACTACCATGTCGGAGATTTCCTATACTGGTTGCAAGAGCACGTGATTGGGTTGGTGT//GCTTGCAATCCAGTGCAATACCTTGCAGCCACTTCCAGAAATCTCCGACACAAGTCTGAT

PlavakaB-1_SLL (Serpula lacrymans)

GL945440.1_[176662-185938]
CGGAGTATCTCATCAGGAAATTGGTGAAAATCTGATCTATGATTGTAAGCCATTATAAGA//GGATTATAAACTCCGCCTGGTAATATCAGCACCTCACCATTTTCCTGATGTCCTTCCTCT
GL945445.1_[289853-280658]
AGCATAATATCATCAGGAAATTGGTGAAAATCTGATCTATGATTGTAAGCCATTATAAGA//GGATTATAAACTCCGCCTGGTAATATCAGCACCTCACCATTTTCCTGATGATAACAGCTA

PlavakaB-2_SLL (Serpula lacrymans)

GL945429.1_[3274237-3267668]
TCTAGACCAGCATCAGTAAAATGTCAACTTTCTTTGAAGACCTTAACTCAGCTTAAAACG//TCAGCAAAGCGTGACCAAGTTCCGTCAAGAAATTTGACACTTTACTGATGCAGGCTGATC
GL945432.1_[2235177-2241812]
GAGTAAGCTACATCAGTAAAATGTCAACTTTCTTTGAAGACCTTAACTCAGCTTAAAACG//TCAGCAAAGCGTGACCAAGTTCCGTCAAGAAATTTGACACTTTACTGATGCTATTGTCCC
GL945432.1_[2867274-2873952]
ATCTTCCAGTCATCAGTAAAATGTCAACTTTCTTTGAAGACCTTAACTCAGCTTAAAACG//TCAGCAAAGCGTGACCAAGTTCCGTCAAGAAATTTGACACTTTACTGATGCACCTGGAGT
GL945435.1_[431775-438367]
TAGTATAAAGCATCAGTAAAATGTCAACTTTCTTTGAAGACCTTAACTCAGCTTATAACG//TCAGCAAAGCGTGACCAAGTTCCGTCAAGAAATTTGACACTTTACTGATGAATAGTGAGA
GL945439.1_[1167205-1173796]
CTCGCAAGGCCATCAGTAAAATGTCAACTTTCTTTGAAGACCTTAACTCAGCTTATAACG//TCAGCAAAGCGTGACCAAGTTCCGTCAAGAAATTTGACACTTTACTGATGAACGCACGCT

PlavakaB-1_SCH (Schizophyllum commune)

GL377302.1_[1515340-1522792]
GGGACATCAACATCAGCATTTTTGGGAGAACCGCATTTGTGCTCGCCGGGGATATGATGT//CGATACATGCCATTACAATCAATCGTGCGGATTTTTAAAATATGCTGATGAAGATGGCCT
GL377310.1_[1480032-1487486]
GCGAGGCAGGCATCAGCATTTTTGGGAGAACCGCATTTGTGCTCGCCGGGGATATGATGT//CGATACATGCCATTACAATCAATCGTGCGGATTTTTAAAATATGCTGATGGGCGAGCTCG

PlavakaB-1_AuSu (Auricularia subglabra)

JH687771.1_[221361-215181]_[221376-215166]
AGATCCCGTGCATCACGAAAACAGGGACGGCGGAAAACGATCCTCTGCCCGCTCTGCGAG//CTGTTAGAGCGCTCAGAGCACAGATTAGCCTTGTCCCTGTTTTCGTGATGTGTCAGAGCT
JH688183.1_[7115-963]_[7116-948]
NNNNNNNNNCCATCACGAAAACGGGGACGGCGGAAAAGCGTCCTCCGTCTGCTCTGAGAG//CTGTTAGAGCGCTCAGAGCACAGATTAGCCGTGTCCCTGTTTTCGTGATGTCCCAGCTTT

Figure S2. Termini and target site duplications (TSDs) of newly characterized *Plavaka* families. TSDs are shown in red. The accession numbers and positions of *Plavaka* insertions are indicated.
